# Development and pilot testing of an online module for ethics education based on the Nigerian National Code for Health Research Ethics

**DOI:** 10.1186/1472-6939-14-1

**Published:** 2013-01-02

**Authors:** Olubunmi A Ogunrin, Temidayo O Ogundiran, Clement Adebamowo

**Affiliations:** 1 Department of Medicine, Neurology Unit, University of Benin, Benin City, PMB 1154, Nigeria; 2 Department of Surgery, University of Ibadan, Ibadan, Nigeria; 3 West African Bioethics Training Program, University of Ibadan, Ibadan, Nigeria; 4 Department of Epidemiology and Public Health, Institute of Human Virology and Greenbaum Cancer Center, University of Maryland School of Medicine, Baltimore, MD, 21201, USA; 5 Office of Research and Training, Institute of Human Virology, 252 Herbert Macaulay Way, Abuja, Nigeria

**Keywords:** Ethics education, Nigerian Code, Online ethics module, Research ethics

## Abstract

**Background:**

The formulation and implementation of national ethical regulations to protect research participants is fundamental to ethical conduct of research. Ethics education and capacity are inadequate in developing African countries. This study was designed to develop a module for online training in research ethics based on the Nigerian National Code of Health Research Ethics and assess its ease of use and reliability among biomedical researchers in Nigeria.

**Methodology:**

This was a three-phased evaluation study. Phase one involved development of an online training module based on the Nigerian Code of Health Research Ethics (NCHRE) and uploading it to the Collaborative Institutional Training Initiative (CITI) website while the second phase entailed the evaluation of the module for comprehensibility, readability and ease of use by 45 Nigerian biomedical researchers. The third phase involved modification and re-evaluation of the module by 30 Nigerian biomedical researchers and determination of test-retest reliability of the module using Cronbach’s alpha.

**Results:**

The online module was easily accessible and comprehensible to 95% of study participants. There were significant differences in the pretest and posttest scores of study participants during the evaluation of the online module (p = 0.001) with correlation coefficients of 0.9 and 0.8 for the pretest and posttest scores respectively. The module also demonstrated excellent test-retest reliability and internal consistency as shown by Cronbach’s alpha coefficients of 0.92 and 0.84 for the pretest and posttest respectively.

**Conclusion:**

The module based on the Nigerian Code was developed, tested and made available online as a valuable tool for training in cultural and societal relevant ethical principles to orient national and international biomedical researchers working in Nigeria. It would complement other general research ethics and Good Clinical Practice modules. Participants suggested that awareness of the online module should be increased through seminars, advertisement on government websites and portals used by Nigerian biomedical researchers, and incorporation of the Code into the undergraduate medical training curriculum.

## Background

The potentials of research to contribute to improvement in health may be surpassed by the magnitude of risk to participants, communities and local researchers. Consequently, systematic ethical regulation of research is put in place to ensure that research is conducted in a manner that maximizes benefits while minimizing harms to research participants. The National Bioethics Advisory Council in USA, the Council of International Organization of Medical Sciences (CIOMS) and the Nuffield Council of Bioethics suggested that this situation needs initial and continuing education in the ethics and science of biomedical and behavioral research for investigators, members of the Research Ethics Committees (RECs), and sponsors of research [1-3]. Several studies have shown that research ethics training improves ethical conduct of research and capacity for dealing with ethical dilemmas [4-6]. Hitherto, researchers in low and middle income countries (LMIC) including Nigeria have had limited access to training in research ethics due to weak educational, social, economic and health resources however there has been significant investment in ethics education in the last two decades with resulting improvements in ethics capacity building [7,8].

The design and implementation of ethically and scientifically valid research in any country should be guided by a set of rules and regulations based on global ethical principles but domesticated within local laws, regulations and culture. It is against this background that the National Health Research Ethics Committee in Nigeria (NHREC) developed and formulated National Code for Health Research Ethics (NCHRE) in August 2007 to guide all researchers involved in human subjects’ researches in Nigeria [9]. This Code reflects the collective concern of the government and the people of Nigeria to ensure the protection of human participants in scientific research to the highest ethical standard that is possible. It serves as the basis for the operations of all institutional research ethics committees in the country thus allowing for uniformity and consistency. The Code is based on universal ethical principles, historical ethical guidelines, existing regulations from different countries, results of modern bioethics research, Nigerian constitution, laws, regulations and government guidelines as well as Nigerian custom and practices.

Examples of peculiarities of the Code include conduct of clinical trials in the country which requires approval from the National Agency for Food and Drug Administration (NAFDAC) and the emphasis on community engagement in research in view of the country’s traditional communitarian ethos. Every scientist involved in human subject research in Nigeria, their sponsors and other stakeholders must function within the scope of the National Health Research Ethics Code. This demands that researchers be aware and educated on the code with the anticipation that the knowledge of the guidelines would translate to its practice in conduct of health-related research.

It is therefore appropriate to develop an educational tool that will facilitate the understanding and implementation of the Nigerian Code among stakeholders. An important avenue for the facilitation of training in research ethics is the provision of online courses [10,11]. Such online courses can be accessed at different institutions in the country and can be effective training tool in research ethics. It will also be of immense benefit to all research ethics committees in the country as it will facilitate education of their members thus empowering them to effectively perform their roles in the protection of human subjects guided by the NCHRE. Furthermore international researchers and foreign sponsors would have easy access to the code which will enlighten them on the ethical requirements for conducting research in Nigeria. There are few studies that have assessed the utility of online modules as ethics training tools in improving knowledge and skills in health research especially in resource poor settings.

It is against this background that this project was conceived and designed with the objectives of developing an online educational module based on the Nigerian Code to enlighten biomedical researchers on the requirements of conduct of ethically acceptable research in Nigeria and evaluate its reliability in achieving this objective.

## Methodology

This was a prospective quantitative three phased study. During phase one, we developed an online training module based on the contents of the NCHRE and upload it to the Collaborative Institutional Training Initiative (CITI) website.

The Nigerian Code is a 68 page document with fifteen main sections (Sections A to O) and one Appendix (with sub-sections 32 to 34) [9]. Because the emphasis of the online module is on ethical requirements for research in Nigeria while avoiding repetition of general ethical guidelines which are already available on the CITI IRB module, we compared the Nigerian Code with thirteen major ethical guidelines, namely; Nuremberg Code [12], Belmont Principle [13], Helsinki Declaration [14], CIOMS [2], United States National Bioethics Advisory Council [1], World Health Organization-Good Clinical Practice [15], World Health Organization- operational guidelines for ethics committees [16], Common Rule – 46 Code of Federal Regulations (CFR) [17], Nuffield Council on Bioethics [3], United Nations AIDS guidance on HIV preventive vaccine research [18], Europeans Medicine Agency – International Committee on Harmonization [19], Opinion of European Group on ethics in science [20] and Directive 2001 [21] and isolated unique elements of the Nigerian Code. These extracted contents formed the material content used to develop our training module (see Appendix A. Annex 1).

The module consisted of 2 parts, an introductory part which enumerated the genesis of the Code and its significance to Nigeria, and the core educational part. The core part contained information on registration of ethics committees, conducting research in institutions without ethics committees, functions and operations of the health research ethics committees, the informed consent process, the health research ethics committee records, continuing ethics training, disciplinary actions against those who violate the Code, the national HREC oversight functions, clinical trials agreement, materials transfer agreement, and other regulatory agencies in Nigeria: including National Agency for Food and Drug Administration and Control (NAFDAC), institutional bio-safety committees, data and safety monitoring board and community advisory board. These elements from the Nigerian Code were thereafter re-written and arranged carefully while ensuring that they did not lose their meanings and interpretations, in the CITI format to make the uploading unto the CITI web site easier. The module was reviewed by two experienced bioethicists who made recommendations on how to improve its presentation without losing the originality of the Code and the revised module was sent to the Director of the CITI for uploading unto the CITI website as a preliminary training module.

### Selection of participants

Forty five research participants were identified purposively and selected by convenience sampling from among senior residents and consultants at the University of Benin Teaching Hospital, Benin, Nigeria, as well as research assistants and medical officers involved in the Presidential Emergency Plan For AIDS Relief (PEPFAR) program at the Institute for Human Virology Nigeria (IHVN), University of Benin Teaching Hospital and Masters of Public Health (MPH) program trainees of the Center for Disease Control, Department of Community Health, University of Benin. All participants had been involved in human subjects’ research. These participants voluntarily consented to participate in the research having received detailed information on what the research was about, what was expected of the participants, the cost to the participant (in terms of time spent to complete the study), expected duration of the task to be performed by the participant, any possible risk (i.e. confidentiality) and anticipated benefits (i.e. knowledge of the Nigerian Code and the award of a certificate on completion of module), incentive to participants (i.e. refreshments during the study), the fact that any of the participants could withdraw from the research at any stage and the address/phone contact of the researcher.

### Evaluation of module

The study participants were allowed to choose a convenient day in the week when they had sufficient time to commence and complete the study. They were divided into three groups based on the day chosen for the pilot testing of the online module and gathered in a relatively spacious, quiet and comfortable room. They were instructed on how to register on the CITI home page, complete the pretest, study the online module and complete the posttest. For new users, access to the module was facilitated by logging onto http://www.citiprogram.org and clicking ‘*New Users – Register Here*’ on the home page. The West African Bioethics Program was selected as training institution in step 1 of the registration procedure and admin@westafricanbioethics.net was entered as the institutional electronic mail address in step 2. On submission of the registration page, a new webpage appeared with the question, ‘Are you conducting human subjects’ research in Nigeria? If you are conducting research in Nigeria, you are required to complete the NIGERIAN NATIONAL CODE FOR HEALTH RESEARCH ETHICS Module’. Access to the module page was granted by indicating ‘YES’. Once registration was completed, subsequent access to the module on CITI home page was obtained by entering username and password.

Each participant thereafter completed the module as instructed. The time taken to study the online module was noted. They were given refreshments during the period of undertaking the study as compensation.

The online module was tested and evaluated for readability and comprehensibility by the 45 study participants during the second phase. The pre-test was a general quiz of multiple choice, true/false 9 questions to test the knowledge of the Code and was administered on paper. The post-test was completed online by the participants and the scores were automatically generated. A survey, with the aid of a questionnaire, was then conducted after the post-test to assess the comprehensibility and the ease of use of the online module, and any other comments on how to improve it were obtained from the study participants. The participants were requested to choose from three options, namely; a) easily accessible or comprehensible, b) difficult to access or comprehend, and c) not accessible or comprehensible. Thereafter the online module was revised based on the suggestions obtained from the survey.

The third phase involved the evaluation of the revised online module. Another set of study participants comprising fifteen participants from the first group and fifteen who did not participate in the initial evaluation assessed the revised module using the same procedure utilized in Phase Two by completing the pretest and posttest on the revised version of the online module. The test performances of the participants in this phase were compared with the performances of the participants in phase two to determine the test-retest reliability of the module.

### Ethical approval

This study protocol was approved by the ethics review committee of the University of Benin Teaching Hospital, Benin City Nigeria. Informed consents were obtained from all research participants. Identifiers were omitted to ensure confidentiality and right of privacy of respondents in line with the ethical principle of autonomy and respect of persons.

### Data management and analysis

The data was recorded in a password protected database, and analysis was done with Stata version SE 10.0 (Stata Corp, USA). The scores from the pre and post tests were analyzed for difference in performance using appropriate statistics for hypothesis testing namely; paired t test (to assess if there was significant difference in means of performances) and correlation coefficients (Pearson product moment). The level of confidence was taken as p values < 0.05. The effects of the demographic variables i.e. sex, age and specialty on pretest and posttest scores were assessed for statistical significance using the likelihood-ratio chi-square analysis. The test-retest reliability of the module was estimated using the Cronbach’s alpha as a measure of internal consistency which ranges from 0 to 1.00. The acceptable level of reliability was 0.80 or higher [22].

## Results

### Demographic details of phase two study participants

Forty five biomedical researchers participated in the initial evaluation of the online module. The participants comprised eighteen females and twenty seven males with a mean age of 35.72 years (SD 3.36) and a range of 25.5 to 45.5 years. The demographic details are as shown in Table 1. Fifteen of the participants were senior resident doctors; seventeen were MPH trainees from diverse backgrounds of health-related practices, nine consultants, two research fellows and two research assistants. Twenty of the study participants (44.4%) had attended any seminar or course on ethics in the past while ten (22.2%) had received training specifically on research ethics. The training were however for short periods – six of the ten who received training in research ethics attended a one day training, two attended a one week training and the remaining two attended a six month training course.

**Table 1:** Demographic data of study participants for phase 2 evaluation

	**Frequency of respondents (N = 45)**	**Percentages**
**Sex**		
**Female**	18	40
**Male**	27	60
**Age**		
**21 – 30**	2	4.4
**31 – 40**	39	86.7
**41 – 50**	4	8.9
**Positions**		
**Consultant/Clinicians**	9	20.0
**Senior Resident Docs**	15	33.3
**Medical Officers**	9	20.0
**Pharmacists**	2	4.4
**Research Assistants**	4	8.9
**Others**	6	13.4
**Specialty**		
**Dental Surgery**	2	4.4
**Family Medicine**	3	6.7
**Internal Medicine**	23	51.1
**Pharmacy**	2	4.4
**Obstetrics/Gynecology**	3	6.7
**Public Health**	5	11.1
**AIDS research (HIV adherence)**	4	8.9
**PMTCT**	2	4.4
**Optometry**	1	2.2
**Location of Research**		
**UBTH**	31	68.9
**University of Benin**	3	6.7
**Irrua Specialist**	1	2.2
**NAUTH Nnewi**	2	4.4
**NNPC Medicals**	1	2.2
**IHVN**	4	8.9
**State HMB**	3	6.7

(Internal medicine specialty includes clinical pharmacology, dermatology, endocrinology, gastroenterology, nephrology and pulmonology).

*UBTH* University of Benin Teaching Hospital*, NAUTH* Nnamdi Azikiwe University Teaching Hospital*, NNPC* Nigerian National Petroleum Company*, IHVN* Institute of Human Virology in Nigeria*, HMB* Health Management Board*, PMTCT* Perinatal Mother To Child Transmission.

### Demographic details of phase three study participants

Thirty biomedical researchers participated in the final evaluation and this group included fifteen participants from the first 45. The group comprised eighteen females and twelve males with a mean age of 34.64 years (SD 4.28) and a range of 25.5 to 45.5 years. The demographic details are as shown in Table 2. Eighteen of them were resident doctors while the rest consisted of five medical officers, two consultants and five research assistants.

**Table 2:** Demographic data of study participants for phase 3 evaluation

	**Frequency of respondents (N = 30)**	**Percentages**
**Sex**		
**Female**	18	60
**Male**	12	40
**Age**		
**21 – 30**	1	3.3
**31 – 40**	26	86.7
**41 – 50**	3	10.0
**Positions**		
**Consultant/Clinicians**	2	6.6
**Senior Resident**	18	60.00
**Medical Officers**	5	16.7
**Research Assistants**	5	16.7
**Specialty**		
**Internal Medicine**	5	16.6
**Public Health**	14	46.9
**HIV/AIDS research**	2	6.6
**IHVN(HIV adherence)**	2	6.6
**PMTCT**	7	23.3
**Location of Research**		
**UBTH**	17	56.7
**University of Benin**	2	6.7
**IHVN**	11	36.6

(Internal medicine specialty includes clinical pharmacology, nephrology, neurology and pulmonology).

*UBTH* University of Benin Teaching Hospital*, IHVN* Institute of Human Virology in Nigeria*, PMTCT* Perinatal Mother To Child Transmission.

### Accessibility and comprehensibility of the online module on Nigerian code

All the participants agreed that the online module was comprehensible and accessible. However 2 of the 45 (4.4%) study participants said that the module was not easily comprehensible. The same pattern was noted for accessibility as 43 indicated that it was very accessible while 2 (4.4%) noted that it was difficult to access. The major challenges of these 2 participants were slow internet speed, irregular power supply and arduous registration procedure.

Twenty three (51.1%) of the 45 completed the online module within 45 minutes while thirty five (77.8%) of the participants completed the module within an hour. Most of 45 participants (97.8%) agreed that the module improved their understanding of the conduct of research in Nigeria. Two participants (4.4%) were not sure if they would recommend the module to someone else.

The responses of the study participants on ways to improve the comprehensibility and accessibility of the module are outlined in Table 3. Forty-two (93.3%) of the respondents suggested that comprehensibility can be improved by increasing awareness of the Nigerian Code among Nigerian biomedical researchers through organization of seminars, distribution of the hardcopies of the Code and the addition of the Code to undergraduate medical curriculum. Twenty (44.4%) of the respondents indicated that accessibility to the module can be improved by introducing the module as a course in tertiary institutions and providing an access link on the Federal Ministry of Health website.

**Table 3:** Suggestions on how to improve accessibility and comprehensibility of the online module based on Nigerian code for health research ethics

**Item**	**Suggestions to improve comprehensibility**	**Frequency of respondents**
**1.**	Design programs and training to create awareness about the Code	26
**2.**	Provide hardcopies of Nigerian Code for researchers	16
**3.**	Add to the curriculum for undergraduate education in Nigeria and make bioethics a core undergraduate course	10
**4.**	Module is voluminous and so should be shortened	6
**5.**	Highlight key issues and definitive statements of interest	4
**6.**	Add more examples and illustrations including pictorial presentations	4
**7.**	Add more information on roles of PIs	4
**8.**	Make webpage more friendly	2
**9.**	Include case scenarios in the module	2
**Item**	**Suggestions to improve accessibility**	**Frequency of respondents**
**1.**	Adequate internet connectivity	12
**2.**	Recommend the module for tertiary institutions in Nigeria	10
**3.**	Provides link for the module on Federal Ministry of Health website	6
**4.**	Make the log in procedure easier for researchers	5
**5.**	Develop a separate website for training on the Nigerian Code	4
**6.**	Encourage computer literacy	2

### Test performance of participants

**Figure 1:**
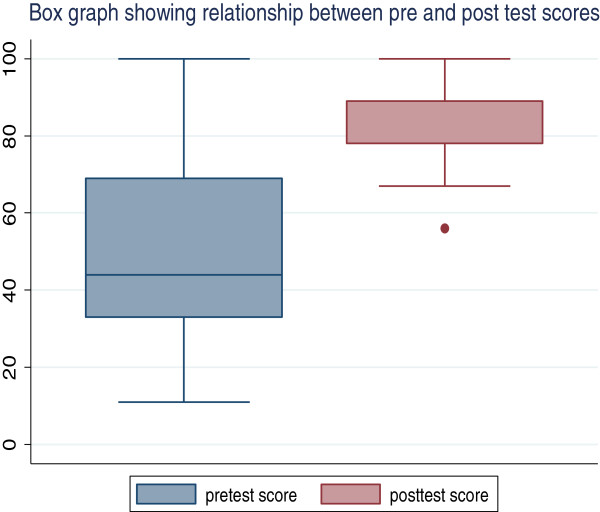
Box Plot showing pretest and posttest scores on initial evaluation of the Module.

All the 45 participants completed the pre-test and post-test for the initial evaluation. The mean time taken to study the online module was 49.8 (SD 15.7) minutes. The mean pre-test score was 53.9 (SD 26.4) and mean post-test score was 83.6 (SD 12.5) which was statistically significantly different (p = 0.001) – Figure 1. The age of the participants did not significantly affect the pre- and post-test scores (p = 0.66). Likewise the sex of the study participants did not affect their scores. The mean pretest scores of the male (N = 27) and female (N = 18) participants were 58.8 ± 28.6 and 57.5 ± 30.9 respectively (p = 0.89) while the means of the posttest scores were 82.2 ± 13.5 and 86.8 ± 8.3 respectively (p = 0.20). The specialty of the study participants did not affect their performances on the evaluation of the online module (p = 0.95). The duration of time taken to complete the pre- and post-tests (p = 0.47) and attendance at any research ethics course (p = 0.23) did not affect their scores.

**Figure 2:**
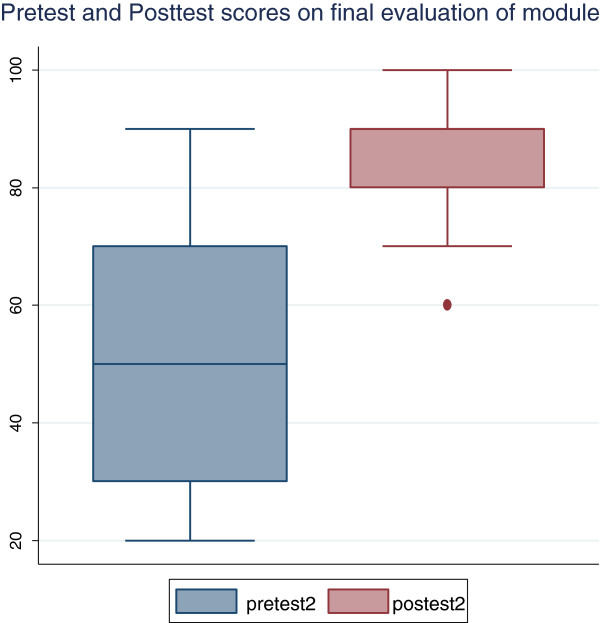
Box Plot showing the Pretest and Posttest scores on final evaluation of online module.

Analysis of the scores of the 30 participants who evaluated the modified version of the module showed a mean pre-test score of 49.3 (SD 21.2) and mean post-test score of 86.0 (SD 11.3) which was statistically significant (p = 0.001) – Figure 2. The age of the participants did not significantly affect the pre- and post-test scores (p = 0.62). Likewise the sex of the study participants did not affect the pretest and posttest scores. The mean pretest scores of the male (N = 12) and female (N = 18) participants were 50.6 ± 20.5 and 47.9 ± 22.6 (p = 0.74) while the means of the posttest scores were 83.1 ± 13.5 and 89.3 ± 7.3 respectively (p = 0.12). The specialty of the study participants did not affect their performances on the final evaluation of the online module with p values of 0.33 and 0.58 for the pre and post-test scores respectively. The duration of time taken to complete the pre- and post-tests (p = 0.58) and attendance at any research ethics course (p = 0.37) did not affect their scores.

### Correlation analysis and reliability testing

The correlation analysis of the pretest scores obtained for the initial and final evaluation of the online module yielded a coefficient of 0.9 and that of the posttest scores yielded a coefficient of 0.8. These coefficients signified excellent correlation between the scores obtained for both the initial and final evaluations of the online module. The test-retest reliability of the test performance on the online module of the Nigerian Code was estimated using the Cronbach’s alpha, a measure of internal consistency of an instrument, by comparing the pretest scores of the initial and final evaluation, and the posttest scores for both evaluations. Values above 0.8 signify acceptable reliability index. The Pretest reliability index was 0.9 while the posttest reliability index was 0.8. The internal consistency of the module as instrument to assess the knowledge of the participants is highly indicative of excellent test-retest reliability index.

## Discussion

This study showed the utility of an online module based on Nigerian Code of Health Research Ethics in improving the knowledge of ethical conduct of research among the study participants as demonstrated by significantly improved scores, satisfactory accessibility and comprehensibility, and excellent internal consistency. The reliability of test instruments is paramount to its utility. An instrument that lacks internal consistency and test-retest reliability is less likely to be effective. The ability of the pretests and posttests on the online module of the Nigerian Code to measure the knowledge obtained from studying the module in a consistent manner is remarkable and compared favorably well with a previous study that validated a new tool for assessment of research ethics knowledge and obtained Cronbach’s alpha of 0.8 [23]. This suggests that the module can be effectively used as a tool for ethics training on Nigerian Code for all researchers conducting biomedical research in Nigeria. This is important for a developing country like Nigeria where ethics capacity is inadequate and the teaching of research ethics is still in its infancy. It is pertinent to stress that the module emphasized the peculiarities of ethical regulations that administrate human subjects’ research in Nigeria hence its focus was not on bioethics as a whole. Therefore it should be used in conjunction with other bioethics and GCP modules. This was the advantage of uploading it onto the CITI website as CITI provides other modules that cover general bioethics training.

A recent randomized study that compared the impact and acceptability of online and onsite methods of training in research ethics and statistics showed that both methods led to marked and similar improvements of knowledge, and that the advantages of less logistical demands and cost-effectiveness made the online method more useful for expanding health research capacity in resource-limited settings [24].

For online trainings, a basic requirement is the availability of internet services. Internet usage has been growing in sub-Saharan Africa in the past decade especially for educational purposes. In Nigeria, all major cities have internet services however quality, bandwidth and consistency is low. Online trainings therefore have to take this into account by avoiding use of media-heavy web pages that require high bandwidth. It is also necessary to ensure web pages resume from previous location in event of service disruption, all of which were factored into the design of this online course.

Furthermore, its access is not limited by subscription as registration on the CITI website is free. The module can be accessed through the West African Bioethics Program institutional portal on the CITI website ( http://www.bioethicscenter.net). This portal is freely accessible to all West African researchers and students. Access to the module would be further enhanced with its inclusion on the website of the Federal Ministry of Health of Nigeria as suggested by some of the research participants. The module has been endorsed by the National Health Research Ethics Committee and its timely update is the responsibility of the NHREC. An endorsed certificate of completion is issued by the Director of the West African Bioethics Training Program on successful completion of the online module. The certificate is sent via electronic mail to the researcher or student.

The most effective way of delivery of ethics education has not yet being clearly identified [25,26] but ethics workshops and conferences have been held in different parts of Africa [8] and there are clinical fellowships on ethics training available in Canada and the United States [27]. One of the suggestions obtained in this study is the inclusion of ethics training in the undergraduate medical curriculum. This is important because ethics education prepares students to address ethical challenges [28], and this multi-faceted formal ethics teaching equip students with a common framework on which to reconcile patients’ medical needs with their values, perceptions, situations and beliefs. It has been noted that the university setting provides an excellent environment for discussions of the principles behind moral reasoning [29]. This is a plausible idea but the shortage of trained bioethicists [8] in sub-Saharan developing nations militates against this.

Now is the time to bridge the ‘ethics education gap’ between the developed and developing countries and resolve the ‘knowledge gap’ in bioethics among researchers in developing countries especially with the increasing trend of multi-national scientific collaborations and conduct of clinical trials in developing countries. This online training tool would therefore serve to orient national and international biomedical researchers working in Nigeria on cultural and societal relevant ethical principles. It is hoped that the knowledge of ethical requirements obtained by studying this module would translate into conduct of ethically acceptable biomedical research in Nigeria. In the nearest future (i.e. 12 months from this evaluation), we planned to conduct assessment of the knowledge and practice of ethical guidelines learnt from the online module among the participants to determine medium and long-term benefits.

## Conclusions

This study has shown that the online module based on the Nigerian Code of Health Research Ethics can be utilized effectively for online ethics education. The performances of the study participants improved significantly on testing after studying the module and the test instrument demonstrated a high internal consistency. As bioethics is being integrated into healthcare settings more widely and systematically, training in bioethics becomes equally important. Online ethics training has gained wide acceptability and many biomedical researchers all over the world had received training in research ethics with the CITI. The addition of this online module to the CITI would ensure accessibility of the Nigerian Code to biomedical researchers interested in doing multi-national research in Nigeria as they study other general bioethics modules on the CITI website, and this in turn will facilitate understanding of particularities of ethical requirements in Nigeria. The final version is online for ethics training at http://www.citiprogram.org.

## Appendix A. Annex 1

### Introduction

All institutions in Nigeria involved in conduct of health research must have a registered health research ethics committee. (See Section C, pp. 16 of the National Code for Health Research Ethics of Nigeria, 2007 and Appendix 1 Section 34 (1) pp. 59 of the National Health Bill passed by the Nigerian National Assembly (Senate) on 15th May 2008).

### Functions of HRECs

Apart from the functions of an ethics review committee which have been covered in other parts of this training program, when a researcher in an institution is conducting research at a location very far from his primary place of employment, he/she will need to find a co-investigator in an institution near the research site and obtain ethical approval from the institution there. For example, a Nigerian researcher who is conducting research in Ghana or Cameroun needs to find a local collaborator and obtain ethical approval from the local institution. The rationale for this is that ethical review of research must be sensitive to local conditions, culture and traditions which only a properly constituted ethics committee in that locality is best positioned to understand and bring to bear on the research process. Furthermore, local authorities must have easy access to a member of the research team in case concerns arise with the conduct of the research.

In Nigeria, an institution may propose to have more than one research ethics committee but the jurisdiction of each must be clearly defined so that there is no overlap. For example, an institution may have one ethics committee for biomedical research, another for social and behavioral sciences research and yet another for animal research. Transparent and open channels of communication between these multiple ethics committees must be put in place so that there is no “ethics committee shopping” by members of staff. Researchers in institutions with more than one ethics committee may not submit the same protocol to more than one ethics committee within the same institution either simultaneously or sequentially. Of course, this does not include situations where a particular committee decides that the protocol is best reviewed by another committee and refers the researchers appropriately. (See Section C, sub-sections c & d, pp. 19 of the National Code for Health Research Ethics in Nigeria).

### Registration of Ethics Committees

In contrast to the practice in many other parts of the world, the regulation in Nigeria requires that ethics committees be registered with the National Health Research Ethics Committee. (See Section C, pp.16 of the National Code for health Research Ethics in Nigeria). The objective of this registration is to ensure that all ethics committees in Nigeria adhere to a minimum standard of composition, management and function, and that they adhere to the tenets of the National Code. A list of all currently approved ethics committees is on the website of NHREC (available at http://www.nhrec.net/). The list contains the names of the institutions, the principal officers of the committees, their contact information, category that the ethics committee falls into and when the registration will expire. Ethics committees are registered for two years only after which the institution shall apply for re-registration. The re-registration application must be submitted to the NHREC within the last six months of the expiry of the current registration.

### Requirements for initial registration

An ethics committee shall be eligible for registration after fulfilling the following requirements:

• Submission of an application package which includes:

○ A letter from the authorized head of the institution or his designee stressing that the line of reporting of the Chairman of the institutional ethics committee is directly to the Chief Executive of the institution. This confers sufficient power on the ethics committee chairman and averts or forestalls undue pressure on the committee in event of disapproval of research protocols,

○ Provision of a list of members of the proposed ethics committee indicating names, qualifications, what profession or status (scientist, non-scientist, lay person, community representative, religious affiliation, gender etc.) they are representing, research review experience of the committee, evidence of completion of NHREC approved research ethics training and any other information that may be construed as conflict of interest on the part of members of the committee;

○ Submission of a statement of agreement to comply with the National Code and,

○ Commitment to provide office and storage space for the committee.

The guidelines for membership selection are similar to those enumerated in international guidelines and documents. Note however that in common with many developing countries, Nigeria is a multi-ethnic, multi-religious society where attention to cultural and religious sensibilities is critically important. (Section C, pp. 16 of the National Code for Health Research Ethics).

### Requirements for re-registration

An institution seeking re-registration for its ethics committee must provide:

• A current list of members, Report of fulfillment of previously stated commitment to provide infrastructure and logistics support for the committee, Complete record of the activities of the committee (including financial records, statutory meetings, complaints and litigations, details of protocols received and their outcomes and the mean time from protocol submission to approval) for the outgoing year.

### What happens if an institution fails to re-register?

The ethics committee is de-registered by the NHREC and the institution has to apply anew. During this period, no research may be conducted in the institution.

Conducting research in institutions without ethics committee (See Section C, sub-section f, pp. 10–11 of the National Code for Health Research Ethics in Nigeria)

In a situation where it is necessary to conduct research in a Nigerian institution or center without ethics committee, there are several options that the researchers may pursue. These include:

• The institution without ethics committee may establish a cooperative agreement with a registered ethics committee located within the same state as the institution or in the event of none in the state, within the same geo-political zone of the country, to review the research. This agreement must be submitted to NHREC for approval and its duration shall not be longer than the registration period of the reviewing ethics committee.

• The researcher may also submit the proposal to NHREC for review. NHREC may review the research using any of several mechanisms that we shall learn about later in this module.

### Functions and operations of HREC

The main objective of institutional ethics committees is the protection of research participants in Nigeria from egregious harm by enforcing compliance with the ethical guidelines in the Code, the clinical trial agreement and material transfer agreement (see below under CTA and MTA). The ethic committees also protect the right of researchers to publish their findings and ensure that they are not exploited or put under undue pressure by sponsors, institutions, participants or communities taking part in research. The committee may demand an agreement, before ethical approval is given, indicating ownership and rights of access to data, resources, intellectual property and infrastructure generated in the course of the research at the pre-research stage to protect the right of researchers to publish their findings and avert future exploitation of the researchers (See Section E, sub-section r, pp. 36–37 of the National Code for Health Research Ethics in Nigeria).

### The review process of research protocols

The review process of research protocols entails the following: (the details of which is available at Section E, pp. 24 of the National Code for Health Research Ethics in Nigeria)

• Shall be conducted at regularly convened ordinary meetings of the institutional ethics committee with simple majority of members present, including at least a member whose primary concerns are in non-scientific areas, except of protocols that require expedited review. In other words, there is no indication for emergency review of protocols with the risk of abuse. Members can participate by phone, video conference or VOIP.

• The ethics committee is expected to complete review of any protocol sent to it within 3 months of receipt otherwise the applicant may complain to the national ethics committee of delay. This provision serves to prevent undue delay in granting approval and commencement of research. Ethics committee that repeatedly delay processing of protocols may find their category downgraded with resultant loss in the types of protocols and research that can be conducted in their institutions.

A researcher may be requested by the ethics committee to pay fees commensurate with the requirements for adequate oversight of research (depending on the size, complexity, duration, status of researcher and sponsor of research) and for any other of its activities at the discretion of the Ethics Committee. Fees can be set only after due consultation with the principal officers of the institution where the ethics committee is located.

• The committee also performs oversight functions for the studies that it has approved to ensure that all stakeholders in research perform their responsibilities.

### Requirements for conduct of research

Research submitted to the ethics committee can only be registered as being properly submitted and accepted for review by the Ethics committee if a complete set of all required documentation has been provided. The minimum requirements are:

• A copy of the research protocol and all materials needed for the consent process. This may include consent form – with translations if necessary, information sheets, radio jingles and video for advertisements, etc.

• Evidence of completion of recognized informed consent training and that of key co-investigators undertaken within two years of the date of submission of application for ethical review of protocol to HREC.

• Brief curriculum vitae (2-3pages) of the principal investigator so that the committee can ascertain his/her qualification to carry out the proposed research.

• Where relevant, copies of letters from co-investigators, laboratories and sources of resources that may be required for the implementation of the project.

• Evidence of sponsorship, where the research is being sponsored by organizations, etc.

• A one page plain language summary of the proposed research

• Copies of all questionnaires, case report forms and instruments to be used in the research.

• Samples of drugs, placebos, medical devices etc. such as may be necessary for the ethics committee to make a decision.

• Decisions of other ethic committees where this is available, for example in multi-centered research

• Copies of all agreements, for example Materials Transfer Agreement (see Section E, sub-section n, pp. 33–34 of the National Code for Health Research Ethics in Nigeria), Clinical Trials Agreement (see section E, of the National Code for Health Research Ethics in Nigeria), Insurance certificates, etc. (Further details are available in the National Code for Health Research Ethics in Nigeria, Section K, sub-section f, pp51-52.)

After approval of research protocol, researchers are required to submit an annual report containing a brief summary statistics of the research (number of research participants, number of adverse events, complaints and their resolutions) to the supervising HREC within three months of expiry of the current research approval.

### Informed consent

Informed consent is an integral part of the ethical conduct of research in Nigeria. All researchers are expected to design a consent process appropriate for the type and context of their research, the expected participants and risks anticipated (See Appendix 1, Section 32, pp. 57 of the National Code for Health Research Ethics).Where consent forms are used, such consent forms must not be longer than 8 pages, typed in double spacing with Times New Roman size 12 font. This is to enhance legibility and prevent researchers from squeezing information into forms. The contents of the consent form should not be beyond the reading ability of a Nigerian Junior Secondary graduate (that is 9 years of formal education) so that participants can understand and recall the contents.The consent form shall contain the following essentially in this order (See Section F, subsection f, pp. 41of the National Code for Health Research Ethics in Nigeria):

• Title of research, Names and addresses of the researchers and sponsors (including telephone, fax, e-mail etc.), The reason for doing the research

• Estimated number of participants in the institution. Where this is a multi-institutional research, the total number of participants should also be stated. Research participants tend to feel more confident if they are part of a larger group than a small one.

• What research participants are expected to do; The expected total duration of research – in months or years. The duration of participants’ involvement. For example, participants may be interviewed for 30 minutes and their blood samples taken; or participants may be interviewed repeatedly over several months for some years.

• The costs to the participants of participating n the research, generally research should not cost participants anything since those participating are doing so out of sense of altruism.

• The risk of participating in the research. This must be presented early in the consent process before introducing information about benefits or compensation so that potential participants are still fresh and engaged when they are given the opportunity to think about the risks that may be involved consequent on their participation.

• The health benefits of participating in research are mentioned next. In many epidemiological studies, particularly of the case–control variety, the cases already have the condition of interest. They are therefore not likely to derive any direct health benefit from participating in the research. However, these information generating researches may improve knowledge about the disease that will benefit others including the controls, family members of cases and even the cases, if the disease may recur in some other parts of their body. Where there is no direct health benefit to participants, this should be clearly stated in the consent form. Compensation for lost wages, transport and opportunity costs as determined by the researchers in consultation with local experts or the ethics committee should then be stated.

• It is sometimes appropriate to give participants inducement in order to encourage their participation in research. What is frowned upon is undue inducement where the amount offered is so large that participants are tempted to carry out actions that are not in their best interest. Usually what constitutes due inducement can be derived from local knowledge and consultation with the ethics committee. With regards to compensation and inducement, the specific amount (or their equivalent in kind) involved should be stated in the consent form.

• The consent form should state the measure that will be used to protect the confidentiality of research participants. This will vary with the sensitivity of the information collected and the complexity of the research.

• The consent form should state clearly and unequivocally that participation in the research is voluntary and include information about what will happen to any individual who chooses not to participate in the research. This usually requires a statement of commitment to the individual that the decision they make will not in any way affect their care in the institution where the research is being conducted. In some situations, the participant may want to withdraw from the research after participating in part or all of it. The mode of dealing with requests for withdrawal in such circumstances varies with the type of research and the individual’s participation. In any event, a clear statement of the modality for orderly withdrawal from the research needs to be included in the consent form.

• Some research participants may suffer injury during participation in research. Because of this, the action(s) to be taken in case of injury or adverse events should be stated. In our environment, this usually requires that the research team takes full responsibility for the care of the research participant once it is established that the injury is a result of participation in the research.

• Increasingly, what happens to individuals or communities when research is over is engaging the attention of ethics committees. This is a complex matter that requires extensive dialogue between researchers, ethics committees, and representatives of the community, the institution and other interest groups. There is no single approach that works in all situations that may arise in research, however careful forethought and planning will avoid future ethical dilemma.

• The consent process should also include adequate disclosure of any apparent or potential conflicts of interest as determined by the ethics committee based on the prevailing norms.

Where written informed consent is not possible (See Section F, sub-section f, p. 43 of the National Code for Health Research Ethics in Nigeria)

In some circumstances, it is not possible to obtain a written consent form. This includes situation where research participants cannot read or are unable or unwilling to read the consent form. In such situations, acceptable options include translating the consent form to local languages where the problem is inability to read English language. In other situations, the participants may be unable or unwilling to read even local languages, in such situations, a witnessed thumb printing or witnessed audio recording of the consent may be done.

### Re-consenting

In some research situations, it may be necessary to take consent more than once. This may be required in situations where participants are being involved in incrementally risky elements of research or in research studies that are taking place over a long duration of time or where information comes to light in the course of a research that changes the risk-benefit analysis of the research project.

### HREC records

Adequate documentation is expected from all registered HRECs in Nigeria. This is particularly needful for the purpose of inspection by the NHREC and if necessary by other agencies through the NHREC, and also in case of future litigation. Documentation of all its activities and materials pertinent to research review which must be kept include:

• Minutes of all meetings which shall contain sufficient information on attendance of members at meetings, actions taken by the ethics committee at the various meetings and how decisions were reached including the vote on decisions, the number of votes for and against and abstentions.

• Letters of complaints from applicants who submitted protocol to the committee. And in event of any change of decision in a proposal status, the reasons for such a change, including the information and/or data used to arrive at the new decision, must be included in the minutes of the meeting at which the decision was taken.

• A written summary of the discussion and how the issues were resolved for issues that are controversial and demanded extensive debates among members.

• All copies of research proposals received and reviewed by the committee, including the reviews from scientific and/or non-scientific experts. These must include the dates proposals were submitted to the committee and when the approvals were given. This provides proof of non-delay in taking decisions on proposals by the committee as it is expected to take decisions within 3 months of receipt of proposal.

• Copies of consent materials approved by the committee (see details above - under ‘Informed consent’)

• All progress reports submitted to the committee by the researchers, institutions and sponsors and these must include details of injuries and adverse events to participants accrue to their participation during and after the research

• All correspondences between the HREC and researchers, sponsors, institutions and all other agents consulted by the committee in the discharge of its duties.

• Details of financial records showing income, direct expenditure and other related expenses.

• Complete records of continuing oversight activities of the HREC for all research protocols approved by it during the period of report. (See Section K, pp 50–52 of the National Code of Health Research Ethics).

### Continuing ethics training and clinics

The Code emphasizes the importance of ethics training and education, a function that may be performed by the institutional HREC or by independent suitably qualified individuals and organizations. Such training programs are acceptable for membership of the HREC and considered adequate training in research ethics for protection of human subjects if they:

• 1. Contain modules on Nigerian National Code of health research ethics, principles of research ethics, HREC functions, research integrity and misconduct, research methodology and administration, and

• 2. Are certified by the NHREC to enforce compliance with ethical principles and guidelines, and avert non-inclusion of NHREC-certified modules in the training curriculum.

For the purpose of aiding researchers in the development of their protocols and providing advisory support during the conduct of research, the HREC may conduct ethics clinics and consultations for a fee at its discretion and as it may determine. This function should however be strictly separated from the HREC oversight responsibility. In addition, suitably qualified individuals or organizations may also offer ethics consultancy services provided they are certified by the NHREC to ensure that there is no departure from the minimum standard expected from all stakeholders in research. (See Sections G, H, I and J of the National Code for Health Research Ethics).

### Disciplinary action (see National Code for Health Research Ethics, Section M pp. 54)

Disciplinary actions would be taken against any researcher, institution or sponsor who is guilty of research misconduct or violates the national code or institutional guidelines. The HREC may recommend such party to the NHREC for disciplinary action after:

• All steps for resolutions of problems in research have been exhausted by the HREC, and

• The actions of the involved party have been fully investigated and discussed at a regularly convened meeting of the committee.

Such recommendation from the institutional ethics committee must reach the NHREC within two weeks following the HREC meeting where the decision was taken. The recommendation must be accompanied by detailed records of the misconduct. It is important to note that if such misconducts are either clear violations of civil and criminal law such as fraud or assault, or of institutional rules, the HREC may report to the appropriate legal authorities or the researcher’s institution as the case may be. The affected researcher, institution or sponsor shall also be duly notified of the HREC decision.

NHREC may in addition take the following actions against any researcher, institution or sponsor found culpable:

• Advertises such cases of research misconducts and report to other appropriate regulatory bodies, and if such breach of ethical conduct involves international collaborative research then the matter is reported to the national regulatory agency of the country of origin of the researcher. This action does not preclude the use of legal action against such researcher or his/her collaborators in Nigeria in accordance with the law. For instance, cases of fraud, deception, fabrication or plagiarism may be reported to the police or other relevant authorities.

• The researcher may be barred from conducting research in Nigeria for a specified period of time based on the severity of his/her offence.

• NHREC may also recommend to institutions and other relevant regulatory bodies penalties that would be faced by researchers who are guilty of misconduct.

• Depending on the case under consideration, the researcher(s) may be required to make restitution to research participants, institutions, sponsors, communities or any other person as directed by the NHREC. For instance, if a researcher is found guilty of deception and fraud, he/she may have to make public apology to the research participants or community involved in research and/or pay back (in cash) the amount involved to either the participants or institution as the case may be and as determined by the HREC or NHREC. In addition, he/she may be barred from conducting research in Nigeria for a period of time.

• In the event that an HREC is the offender, then the HREC may be suspended for a variable period of time based on the severity of offence.

### NHREC oversight functions

The National Health Research Ethics Committee may review research protocols:

• If it involves nationwide trial (more than 3 research sites). For example the protocol of a trial to determine the clinical efficacy of a drug that is being introduced into the Nigerian market which is to be tested among patients attending four tertiary health facilities in different parts of the country requires NHREC review; or

• If it is referred by an institutional ethics committee either because the HREC is not qualified to review or otherwise; or

• In cases of institutions where no ethics committee exists and such institution has no cooperative agreement with another HREC (as stated above under Conducting research in institutions without ethics committee); or

• If the NHREC so desires based on the complexity of the protocol, the magnitude of risks and existence of controversies like issues of standard of care. The NHREC may achieve this by either mandating an institutional ethics committee (thus acting as an HREC of record) to review implying that such committee takes over the oversight function, or the NHREC may constitute itself into a committee for the purpose thus retaining the oversight function. (See Section L, pp. 53 of the National Code for Health Research Ethics for details)

The NHREC also reviews the annual report and registration status of the HRECs, and institutions’ commitment to ensure proper functioning of the HRECs. It also settles disputes arising from appropriate interpretation of the Code as it is the NHREC that is responsible for the update, revision and modification of the Code in accordance with new developments in international research ethics.

In addition, the NHREC establishes the categories of HRECs in the country on the basis of the following criteria (See Section C, subsection g, pp. 20 and Appendix 1, Section 34, pp. 59–60 of the National Code of health Research Ethics):

• Size of the committee and the qualifications, training and experience of its members in research ethics and science. The more qualified and experienced the members of a committee are, the higher the rating accorded the HREC by the NHREC. This criterion is not taken in isolation but with others stated below.

• History of the committee vis-à-vis its past review activities from records submitted by the HREC to the NHREC, the period that the committee has been in existence, how good its record keeping has been and the level of its compliance with the National Code.

• The amount of resources at the disposal of the committee and the affiliated institution i.e. supporting personnel, infrastructure and finances.

These criteria are clearly outlined from time to time so that all HRECs are aware of the pre-requisites for rating them by the NHREC at the latter’s regularly scheduled meetings. Each category of HREC in Nigeria shall be assigned the types of research it can review. This implies that some HRECs may not be qualified to review some research protocols based on their categorization by the NHREC. Such protocols may be reviewed by qualified HREC within the same institution or same geo-political zone or by the NHREC.

There are two important agreements that ensure adequate protection of research participants and the research staff in Nigeria. These are clinical trial and material transfer agreements.

### What is the clinical trial agreement (CTA)?

• It is a contract and agreement between a research sponsor and the principal investigator (PI) of a clinical trial enforcing compliance with all ethical regulations governing conduct of research in Nigeria. This agreement is signed by the PI, the research sponsor and head of institution (i.e. where the ethics committee reviewing the protocol is sited).

• It is a statement of commitment made by the principal investigator to conduct trial in compliance with the National Code and the ICH-GCP (Available at http://www.ich.org/ ), and/or ISO 14155 for trials of medical devices and also in compliance with guidelines from relevant oversight and regulatory agencies, institutional guidelines and Federal Ministry of Health requirements.

• This agreement also demands that the principal investigator and his/her institution where the ethical approval is obtained will cooperate fairly and appropriately in the event of legal claim relating to the conduct of trial. This implies that there shall be amicable resolution of issues arising from the research, for instance the institution may assist in the care of research participants who suffer some form of injury or adverse event in the course of experimentation, or in the event of litigation from community participants, and not leave the researcher alone to carry the responsibility,

• In the event that the research sponsor is the PI, then he/she must include in the agreement the estimated cost of and sources of funds for the trial, the number of participants, and a complete budget of the trial and the dates of payments.

### What is the material transfer agreement (MTA)?

• It is a mandatory agreement that is required if a research involves the transfer of samples and biological materials such as animals, herbs and plants out of Nigeria and it must be signed by all parties involved in the research including the local and international investigators, heads of local institutions, and research sponsors. The rationale for this agreement is to protect the (a) local researchers and Nigeria’s human and natural resources in all its biodiversity, (b) community participants in research and (c) the nation from exploitation and harm.

• The agreement must contain the details and conditions for the transfer of the samples such as:

(a) The type of materials that is to be transferred and its anticipated use, (b) The locations and duration of storage of the material outside Nigeria, and (c) The limitations to the use of such materials and when its use will be terminated.

• The MTA must be reviewed by the HREC to ensure that it conforms to the objectives of the research and contents of the informed consent. The institutional REC may grant provisional approval for transfer of samples pending the submission of both the MTA and provisional approval of the HREC to the NHREC who will acknowledge its receipt for record purposes only. It is the responsibility of the researcher to inform the institutional HREC of the NHREC’s acknowledgement of receipt of the MTA. This prevents undue delay in the commencement of research but this does not preclude withdrawal of approval by the HREC or the right of participants or communities from whom samples are obtained to withdraw them according to terms of the informed consent process. Thus ensuring the preservation of their autonomy and demonstration of respect for persons in research.

• The institutional HREC has the responsibility to grant final approval to research involving the transfer of research samples out of Nigeria. But such approval can only be given if all the other criteria as stated in the National Code or institutional guidelines have been met and the NHREC has acknowledged receipt of the MTA.

• In the event that the NHREC fails to acknowledge receipt of the MTA in 2 weeks and there is a verifiable proof that the applicant has sent same to the NHREC, then the institutional REC can issue the final approval for the research.

• If the researcher or PI makes any amendment to the MTA, he/she is expected to submit a request for amendment of protocol to the institutional HREC and the HREC shall review it in the usual manner as for protocol amendment at its regularly convened meeting.

(See details at Section E, subsection n, pp 33–34 of the National Code for Health Research Ethics).

### Other Nigerian regulatory agencies in research

The conduct of clinical trials in Nigeria requires the approval of other regulatory agencies apart from the institutional ethics committee. There are four regulatory agencies specified in the National Code that contribute to protection of research participants from harm and ensure conduct of trials at the highest ethical standard.

### National Agency for Food and Drug Administration and Control (NAFDAC)

• This agency gives permission for the conduct of clinical trials to test the efficacy and safety of new finished products for sale or use as food or drugs in Nigeria.

• Such clinical trials that would be conducted in Nigeria must however conform to the guidelines issued by National Health Ethics Research Committee and other regulatory bodies.

### Institutional Bio-safety committees (IBC)

• The IBC provides bio-safety review of research involving the use of classified hazardous substances of physical or biological nature (like pathogens, radioactive materials, application of recombinant DNA techniques and processes etc.) with the overall objective of minimizing potential human and environmental risks.

• It is made up of a bio-safety officer and at least three other officers with appropriate expertise, and is established by the institution where research is conducted and registered with the NHREC (see NHREC oversight functions above)

• The IBC ensures that the researcher provides safe and suitable storage for materials used for research and also ensures that research staff has adequate training in bio-safety.

### Data and Safety Monitoring Boards (DSMB)

Data and Safety Monitoring Boards (DSMB) – is an independent group of experts (comprising individuals with appropriate training and scientific knowledge in all aspects of research with adequate medical, pharmaceutical, scientific, bio-statistical and clinical trial experience as well as ethics qualifications) assembled before the commencement of research by the study sponsors to review safety data during the clinical trial.

At least three of the members must be independent of the clinical trial and sponsor. This is to avert or prevent conflicts of interests and allow for unbiased assessment of safety data.

All drug efficacy trials and clinical trials in Nigeria are expected to have a safety monitoring plan implemented through the DSMB to ensure safety of study participants and preserve the integrity and credibility of the research.

### Community Advisory Board (CAB)

This committee is established by the study investigators on the recommendation of the institution or the ethics committee supervising the trial depending on the nature and site of research. The role of community ‘gatekeepers’ in the successful implementation of research in Nigeria underscores the importance of the CAB. The multi-ethnic, cultural and religious diversities of the Nigerian nation and its influence on ethical issues in research demand community participation from the pre-research stage to avoid conflicts and frictions during and after the research.

The CAB consists of members, from the communities where trials are to be conducted, who are selected through the usual consultative process with the community (for example religious leaders, persons who understand local laws and cultural values including gender issues, representative of study population, and community leaders) and some members of the research team (usually not more than 20% of the total membership).

The CAB plays crucial roles in assisting researchers conducting trials in Nigeria to understand and incorporate community concerns into their research plans and activities. It provides information on traditional beliefs; advises on effective methods of information dissemination, recruitment and retention of study participants and may assist in the resolution of ethical problems that arise during and after research (See Section E, subsection r, pp. 37–38 of the National Code of Health Research Ethics).Furthermore, the CAB offers the community members to air their views on ethical issues relating to the proposed research as they affect individual members, the community as a whole, and possibly neighboring communities and the nation. The Board also serves as s forum for disseminating pre, intra and post research information to members of the community.

(details of the Nigerian Code of Health Research Ethics can be accessed at http://www.nhrec.net).

## Competing interests

The authors have no conflicting interests to declare.

## Authors’ contributions

CA conceived of the study. OAO, TOO and CA participated in the design and coordination of the study. OAO collected data, performed the statistical analysis and helped to draft the manuscript. All authors read and approved the final manuscript.

## Pre-publication history

The pre-publication history for this paper can be accessed here:

http://www.biomedcentral.com/1472-6939/14/1/prepub
